# From Foundations to Frontiers: Best Papers in Bioinformatics and Translational Informatics 2024

**DOI:** 10.1055/s-0046-1816605

**Published:** 2026-11-03

**Authors:** Scott McGrath, Mary Lauren Benton

**Affiliations:** 1 CITRIS Health, University of California BerkeleyUSA; 2 Department of Computer Science, Baylor UniversityUSA

**Keywords:** Bioinformatics, Genomics, Transcriptomics, Machine Learning, Precision Medicine, Multi-omics, Foundation Models, Antimicrobial Peptides

## Abstract

**Objectives:**

To identify and summarize the top bioinformatics and translational informatics (BTI) papers published in 2024, we focus on advancements in disease prediction, foundation models for single-cell biology, and antimicrobial discovery.

**Methods:**

We conducted a comprehensive literature search using PubMed and BlueSky crowdsourcing to identify candidate BTI papers. The 620 resulting articles were systematically reviewed by section editors using three primary criteria: novelty, significance, and quality. A final list of 15 candidate papers was then evaluated by external reviewers to select the top three papers for this year.

**Results:**

Three outstanding BTI papers demonstrated significant contributions to the field. These papers showcase innovative approaches to leverage machine learning for disease prediction using multi-omics data, develop foundation models for single-cell biology, and apply computational methods to identify novel antimicrobial peptides from the global microbiome. In addition, our honorable mention paper provides a framework for building a virtual cell using artificial intelligence (AI).

**Conclusions:**

This year’s top BTI papers reflect several important trends, including the integration of multi-omics data for improved disease prediction, the application of foundation models to biological data, and the use of machine learning to address critical healthcare challenges such as antimicrobial resistance. These papers highlight how computational approaches continue to drive innovation in precision medicine and biological research

## 1. Introduction

In 2024, the scientific community witnessed remarkable advancements in bioinformatics and translational informatics (BTI), building upon the foundation of previous years’ achievements. As digital technologies continue to transform healthcare and biomedical research, there is an increasing emphasis on developing comprehensive computational models that can integrate diverse biological data types to enhance disease prediction, treatment selection, and drug discovery. The integration of artificial intelligence, particularly transformer architectures, has driven significant progress in these domains, enabling researchers to extract meaningful insights from complex biological datasets.

In this increasingly data-rich environment, bioinformatics and translational informatics play critical roles in bridging the gap between basic biological research and clinical applications. The complex nature of biological systems requires sophisticated computational approaches to analyze, interpret, and translate high-dimensional data into actionable insights for healthcare providers and researchers. This year’s top papers showcase innovative solutions to these challenges, demonstrating the power of computational approaches in advancing our understanding of human health and disease.

The papers selected for this review exemplify several important trends in the field. First, we observe continued development of machine learning frameworks capable of integrating diverse data types, including genetic, proteomic, and clinical information, to improve disease prediction. Second, there is growing interest in foundation models for biological data, paralleling developments in natural language processing and computer vision. Third, we see novel applications of computational methods to address pressing global health challenges, such as antimicrobial resistance. Finally, there is increasing attention to the development of virtual cellular models that can simulate complex biological processes.

In this synopsis, we present the methodological approach used to identify the top BTI papers of 2024, describe the major themes from our candidate best papers, and discuss their implications for the future of bioinformatics and translational informatics.

## 2. Methods

To identify the top bioinformatics and translational informatics papers published in 2024, we implemented a comprehensive and systematic approach to literature search and evaluation.

### 2.1. Literature Search

We conducted an extensive literature search using PubMed with a specific focus on papers published in 2024. Our search strategy employed a combination of Medical Subject Headings (MeSH) terms and keywords related to bioinformatics, computational biology, and translational informatics. The search query was designed to capture papers at the intersection of computational methods and biological applications:

**Table:** 

Search Query
(“bioinformatically”[All Fields] OR “computational biology”[MeSH Terms] OR (“computational”[All Fields] AND “biology”[All Fields]) OR “computational biology”[All Fields] OR “bioinformatic”[All Fields] OR “bioinformatics”[All Fields] OR “translational research, biomedical”[MeSH Terms] OR “algorithms”[MeSH Terms] OR “machine learning”[MeSH Terms]) AND (“genetics, medical”[MeSH Terms] OR “genomics”[MeSH Terms] OR “gene expression profiling”[MeSH Terms] OR “transcriptome”[MeSH Terms] OR “proteomics”[MeSH Terms] OR “epigenomics”[MeSH Terms] OR “metagenomics”[MeSH Terms] OR “omics”[All Fields] OR “precision medicine”[MeSH Terms] OR “precision medicine”[All Fields]) AND (“humans”[MeSH Terms])

This query was designed to identify papers that combined computational approaches with biological data types relevant to human health. We specifically focused on articles published in high-impact journals in the field, including but not limited to: Journal of the American Medical Informatics Association (JAMIA), Journal of Biomedical Informatics (JBI), PLoS Computational Biology, Bioinformatics, BMC Bioinformatics, BMC Systems Biology, Nature, Nature Genetics, Nature Biotechnology, Nature Methods, Science, Science Translational Medicine, Clinical Pharmacology and Therapeutics, New England Journal of Medicine, Journal of the American Medical Association (JAMA), Lancet, PLoS Genetics, and Cell.

In addition to our structured PubMed search, we also utilized BlueSky crowdsourcing to identify potentially relevant papers that might have been missed by our search strategy. This approach helped ensure comprehensive coverage of the literature and minimized the risk of overlooking significant contributions to the field.

### 2.2. Screening and Evaluation Process

Our search yielded a total of 620 articles, which were systematically screened and evaluated in a multi-step process:

(i) Initial screening: the section editors conducted an initial review of all 620 articles using Rayyan, a web-based tool designed to facilitate systematic reviews. Articles were screened based on their titles and abstracts to determine their relevance to the field of bioinformatics and translational informatics;
(ii) Detailed evaluation: articles that passed the initial screening were subjected to a more detailed evaluation based on three primary criteria:
  - Novelty: the originality and innovation of the research approach or methodology;
  - Significance: the potential impact of the work on the field of bioinformatics and translational informatics, as well as its relevance to healthcare applications;
  - Quality: the technical soundness of the work, including methodology, data analysis, and interpretation of results;
(iii) Selection of candidates: based on the detailed evaluation, a final list of 15 candidate papers was selected for further review;
(iv) External review: the 15 candidate papers were uploaded for review by external experts in the field, who evaluated each paper based on its importance, scientific/practical impact, scientific content, originality, quality of literature, and quality of presentation;
(v) Final selection: based on the combined scores from the section editors and external reviewers, the top three papers were selected for inclusion in this year’s review, along with one honorable mention paper.

Throughout this process, we aimed to identify papers that not only demonstrated technical excellence but also had significant potential for translational impact in healthcare and biomedical research.

**Table 1:** Selection of best papers for the IMIA Yearbook of Medical Informatics for the Bioinformatics and Translational Informatics section. Articles are listed in alphabetical order of the first author’s surname (see summary in Appendix). * denotes the honorable mention. country.

Section Bioinformatics and Translational Informatics
*Bunne C, Roohani Y, Rosen Y, et al. How to build the virtual cell with artificial intelligence: Priorities and opportunities. Cell. 2024 Dec 12;187(25):7045-7063. PMID: 39672099. https://doi.org/10.1016/j.cell.2024.11.015 . Cui H, Wang C, Maan H, et al. scGPT: toward building a foundation model for single-cell multi-omics using generative AI. Nat Methods. 2024 Aug;21(8):1470-1480. https://doi.org/10.1038/s41592-024-02201-0 . Garg M, Karpinski M, Matelska D, et al. Disease prediction with multi-omics and biomarkers empowers case-control genetic discoveries in the UK Biobank. Nat Genet. 2024 Sep;56(9):1821-183. https://doi.org/10.1038/s41588-024-01898-1 . Santos-Júnior CD, Torres MDT, Duan Y, et al. Discovery of antimicrobial peptides in the global microbiome with machine learning. Cell. 2024 Jul 11;187(14):3761-3778.e16. https://doi.org/10.1016/j.cell.2024.05.013 .

## 3. Themes

From our comprehensive evaluation process, we identified three outstanding BTI papers published in 2024, along with one honorable mention. These papers exemplify the cutting-edge research being conducted at the intersection of computational sciences and biomedicine. Here, we discuss a few themes from our candidate best papers.

### 3.1. Foundation Models for Applications in Biology

Foundation models have seen a meteoric rise in use and interest, largely owing to the popularity of large language models. Foundation models are a class of deep learning models, typically using a self-attention transformer architecture, trained on massive datasets and designed to complete a wide variety of tasks. In bioinformatics, these tasks include things like prediction of -omics profiles, cell type or cell state annotation, and multi-omic integration. A necessary component for foundation models is the training dataset. As discussed by Rood et al. [ 1 ], cell atlases such as the Human Cell Atlas (HCA) will provide comprehensive reference maps of all human cells that will inform AI training. Atlases like HCA are becoming increasingly informative thanks to advances in experimental techniques such as single-cell and spatial -omics sequencing as well as larger sample sizes. Ideally, these atlases will cover a wide range of tissue and cell types, developmental stages, diseases states, and molecular scales to better power downstream modeling efforts.

Two foundation models introduced in 2024 were included among our best paper candidates. Fu et al. [ 2 ] trained the general expression transformer (GET) on chromatin accessibility and sequencing data from more than 200 fetal and adult cell types. GET was able to accurately predict gene expression levels across cell types and identify both gene regulatory elements and their interactions. Experimental assays to complete these tasks can be challenging and expensive to perform, so in silico predictions can inform researchers about the links between genome sequences and transcriptional output across tissues. The other foundation model was introduced by one of our best papers: single-cell generative pretrained transformer (scGPT) [ 3 ]. The scGPT model was trained using single-cell RNA-sequencing from more than 33 million cells. The diverse applications of foundation models are highlighted in the paper, where fine-tuned versions of scGPT model were shown to outperform the current state-of-the-art on six common downstream analysis tasks: cell type annotation, genetic perturbation effect prediction, batch correction, multi-omic integration, clustering, and gene-network inference.

We are only beginning to unlock the potential of foundational models to transform how we use existing datasets and advance bioinformatics research. To this end, our honorable mention, Bunne et al. [ 4 ], proposed a vision for how we can integrate foundational models across biological scales—from molecular and phenotypic data to spatial information—to develop a “virtual cell”. By creating virtual models of cellular function using AI, future researchers would be able to conduct increasingly complex experiments in silico and guide the next generation of experimental research. Although many challenges remain before the dream of a virtual cell can be realized, advances like the ones discussed in this section are bringing us closer to creating integrated, dynamic representations of cellular systems.

### 3.2. Computational Methods to Build Large-scale Biological Data Resources

The role of machine learning in biological research has evolved significantly, shifting from primarily analytical applications toward the systematic construction of comprehensive biological data resources. This represents a fundamental change in how we approach large-scale biological discovery and resource development. This year, several of our candidate papers both develop data resources and leverage them to answer pressing questions related to global and human health. In a best paper, Santos-Junior et al. [ 5 ] use random forest models to predict antimicrobial peptides (AMPs), applying the models to tens of thousands of metagenomes and microbial genome sequences. They turn the resulting 863,498 non-redundant peptide sequences into AMPSphere, an open-access database that can shed light on the evolutionary origins of AMPs and improve antibiotic discovery. The scale of this effort, moving from scattered individual discoveries to systematic, machine-guided exploration, represents a new paradigm for biological resource development. Similarly, Jee et al. [ 6 ] develop MSK-CHORD, a data resource for cancer research derived from free-text clinical, radiology, and histopathology notes, in addition to traditional structured clinical and genomic data. To increase the project scale, the authors applied a transformer-based natural language processing model to perform automated annotation of free-text notes before integrating these features with structured data. The resulting data resource includes information on more than 24,000 patients across five cancer types and was used to train machine learning models to predict patient survival.

Other papers demonstrate the usefulness of traditional machine learning frameworks when applied to novel experimental datasets. For example, Deng et al. [ 7 ] applied machine learning approaches to create a comprehensive plasma proteome atlas, mapping 2,920 proteins across hundreds of diseases and health-related traits in a cohort of over 53,000 individuals. The authors applied clustering and other statistical analyses to understand the biological underpinnings of these associations and identify potential therapeutic targets. The resulting open-access resource provides researchers with unprecedented insight into disease mechanisms and potential biomarker discovery pathways. Eteleeb et al. [ 8 ] focused on neurological applications, using machine learning to integrate high-throughput multi-omics data from human brain cohorts to identify distinct molecular profiles associated with Alzheimer’s disease. They discovered four clusters, where the features of one cluster were specifically associated with poorer disease outcomes and enriched for biological pathways dysregulated in Alzheimer’s disease. These developments suggest that machine learning is becoming less of an analytical tool and more of a foundational infrastructure for biological research. However, the success of these initiatives will ultimately depend on the research community’s ability to effectively utilize these resources and integrate them into clinical practice. The challenge ahead lies not just in building these comprehensive databases, but in ensuring they translate into meaningful advances in patient care and therapeutic development.

### 3.3. Integrative Machine Learning for Disease Insights

As in previous years, advances in machine learning methodologies continue to be applied to biological data to generate new, translational insights. One of our top papers developed MILTON, an ensemble machine-learning framework that uses biomarkers to classify individuals by disease status [ 9 ]. MILTON has the potential to improve disease classification because the model first uses known cases/controls to develop a disease signature; it then uses that signature to identify previously unknown or cryptic cases among the control cohort. These improved case/control cohorts can then be used in downstream genetic association analysis. MILTON was better able to predict disease diagnosis than traditional polygenic risk scores in most cases (111/151 ICD10 (International Classification of Diseases 10) codes tested) and was even able to prospectively predict case status using data collected prior to disease onset. In general, cohorts augmented using MILTON were able to replicate known gene-disease associations, and the increase in statistical power resulted in 182 novel gene-disease associations. Because the approach is based on a relatively small set of plasma biomarkers, methods such as MILTON could provide important information for early disease prediction.

Three other candidate papers also introduced new computational approaches to classify clinically relevant outcomes including disease state, prognosis, and treatment outcomes. Liu et al. [ 10 ] developed Pathformer, a model based on the popular transformer architecture, that gains biological relevance by encoding gene features along with biological pathway information. When applied to data from The Cancer Genome Atlas (TCGA), Pathformer generally outperformed traditional machine learning architectures as well as other deep learning models. On average, the F1 scores improved between 5-14% when predicting cancer survival, cancer stage, and drug response. Keyl et al. [ 11 ] also developed a model to predict cancer outcomes but focus on the explainability of their machine learning model to increase clinical relevance. Importantly, this study focused on assessing the contributions of model features to performance on the patient level, highlighting markers important for individual patients rather than patient populations. Finally, Wang et al. [ 12 ] introduced HyperTMO, a hypergraph convolutional neural network model that they used to classify patients (cancer subtype for breast cancer patients, case/control status for Alzheimer’s disease). In HyperTMO, hypergraphs were built from different types of multi-omic data—in this case, mRNA, miRNA, and DNA methylation—before constructing a hypergraph convolutional neural network for each -omic hypergraph and performing late-stage integration.

### 3.4. Addressing Limitations in Current Genome-wide Analyses

In addition to the development of complex machine learning architectures, two of the candidate best papers explore technical limitations in the genome-wide association studies (GWAS). GWAS are still widely used in genomics, and access to large biobank cohorts has greatly improved their statistical power over the last 10 years. However, phenotype missingness can impede these studies, so recent work has leveraged machine learning (ML) to impute phenotypes before performing GWAS. Miao et al. [ 13 ] explored this trend and found that ML-assisted GWAS can result in a high false positive rate. To mitigate the false positive rate, they propose the Post-Prediction Genome-Wide Association Study (POP-GWAS), an alternative that computes SNP-level effects on both the imputed and observed phenotypes. It uses the difference between these effects to adjust the overall estimate of SNP effects. This approach improves the validity of ML-assisted GWAS while improving the statistical power. Furthermore, by requiring only GWAS summary statistics for POP-GWAS, this approach could be more widely applied to improve the accuracy for future ML-assisted GWAS.

GWAS-by-proxy (or GWAX) have also become popular for certain phenotypes, as they allow researchers to use parental diagnoses as a proxy phenotype for GWAS rather than requiring a direct patient diagnosis. This is particularly relevant for neurodegenerative diseases such as Alzheimer’s disease, where diagnoses are often made late in life and are not yet captured in the biobank data. In their paper, Wu et al. [ 14 ] highlight major limitations of GWAX, where they show that meta-analyzing over parental phenotypes identifies many loci that do not agree with direct GWAS results and induces false genetic correlations with educational attainment and cognition-related phenotypes. They explored several options to mitigate these biases; however, none were completely sufficient. Considering these results, the authors urge caution when conducting future GWAX and suggest that summary statistics for GWAS and GWAX should be reported separately.

## 4. Conclusion

The top BTI papers selected for 2024 demonstrate the diverse and innovative approaches being applied to solve complex problems in biology and medicine through computational methods. Overall, we find that the advances in multi-modal data collection and increasingly large datasets lay the groundwork for more complex computational modeling. In particular, the growth of foundation models in biology has the potential to revolutionize how we interpret complex biological data, discover the molecular underpinnings of disease, and enable more precise approaches to diagnosing and treating patients.
